# Transitional care for young adults with ADHD: transforming potential upheaval into smooth progression

**DOI:** 10.1017/S2045796019000817

**Published:** 2020-01-09

**Authors:** Tamsin Ford

**Affiliations:** Department of Psychiatry, University of Cambridge, Douglas House, 18B Trumpington Road Cambridge CB2 2AH, Exeter, UK

**Keywords:** Access to treatment, ADHD, service organisation, transition

## Abstract

Increasing numbers of young adults need continued support for their attention deficit hyperactivity disorder (ADHD) beyond the age-boundary for children's services. The sparse literature on transition in general suggests patchy provision and huge gaps in transitional care, but also that young people with ADHD and other neurodevelopmental disorders fair particularly badly. Transition in health care coincides with many other important life-transitions while the difficulties associated with ADHD may make these challenges particularly hard to cope with. Parents or other advocates therefore often need to be involved, which can present problems in adult mental health services given that they tend to be less family oriented than children's services. Importantly, young people need help negotiating the transition from passive recipient of care to active self-management, and in building relationships with the adult team.

In addition to patchy provision of adult ADHD services, transition is currently hampered by poor understanding of ADHD as a long term condition and uncertain knowledge of what services are available among young people and parents as well as the clinicians working with them. Guidelines recommend, and more importantly young people want, access to psycho-social interventions as well as medication. However, available evidence suggests poor quality transitional care and adult services that are highly focused on medication.

Adult ADHD services need to undergo similar development to that experienced by Child and Adolescent Mental Health Services and community paediatrics over the last few decades. While we debate the relative merits of dedicated or specialist *v*. generic adult mental health services, for young adults with ADHD the training, experience and availability of professionals are more important than their qualifications or setting.

Attention deficit hyperactivity disorder (ADHD) is a common reason for attendance at Child and Adolescent Mental Health Services (CAMHS) or community paediatrics (Ford *et al*., 2007). A meta-analysis of 41 population-based mental health surveys of children under the age of 18 years (*n*-87 742) estimated the prevalence to lie between 2.6 and 4.5% (Polanczyk *et al*., 2015). A varying but small proportion of these children access services (Ford *et al*., 2007), but rates of clinical diagnosis and prescriptions for stimulant medication in childhood have steadily increased during the last 40 years in many parts of the world as a result of better recognition and improved service provision (Coghill, 2017). While initially conceptualised as a disorder of childhood, 15% of those affected during childhood continue to meet full diagnostic criteria for ADHD into their mid-twenties, and a further 50% continue to struggle with impairment due to sub-clinical symptoms (Faraone *et al*., 2006). The result is an expanding cohort of young adults who need access to treatment.

The sparse literature on transition between child and adult mental health services in general suggests that it is ‘poorly planned, poorly executed and poorly experienced’ (Singh *et al*., 2010; Signorini *et al*., 2018; Appleton *et al*., 2019). Evidence suggests that those with neurodevelopmental conditions, such as ADHD are particularly likely *not* to transfer to adult services (Singh *et al*., 2010; Buitelaar, 2017; Tatlow-Golden *et al*., 2018; Appleton *et al*., 2019). A little more than a decade after the need was formally recognised, this editorial describes what we know about transitional care among young adults with ADHD to identify progress and gaps (Nutt *et al*., 2007).

## Transition is a process

Adolescence involves major developmental changes and challenges, while transitions, even if ultimately positive, require a period of adaptation. Within the context of healthcare, the consensus is that transition should extend beyond the simple transfer of clinical responsibility with an aim to support a young person into a new life-stage in a way that optimises their health and function (Beresford, 2004). The timing of the transfer between child and adult services often coincides with other major life transitions, such as leaving education, starting work or moving out of the parental home (Cleverley *et al*., 2018). Thus, young adults may be deprived of familiar support networks while facing increasing demands in several domains simultaneously (Singh *et al*., 2010; Signorini *et al*., 2018). The constellation of difficulties that comprises ADHD may be particularly salient to transition; reduced ability to organise and regulate the self, as well as the high levels of comorbidity seen in clinical populations makes coping with these changes especially challenging, while impaired function during this critical and rapid developmental period is particularly detrimental to health, educational, occupation and social outcomes (Young *et al*., 2016; Buitelaar, 2017; Janssens *et al*., in submission). National registry studies suggest that continued ADHD medication into early adulthood is associated with substantially reduced levels of suicidal behaviour (Chen *et al*., 2014), depression (Chang *et al*., 2016), substance misuse (Chang *et al*., 2014), road traffic accidents (Chang *et al*., 2017), convictions and violent reoffending (Lichtenstein and Larsson, 2013). Transitional care for the graduates of children's services with ADHD and ongoing clinical need is, therefore, extremely important.

At its simplest, optimum transition has been characterised by *planning, information transfer* between the referring and receiving teams, *joint working* and most importantly, *continuity of care* (Royal College of Paediatric and Child Health, 2003; Singh *et al*., 2010; NICE, 2016). There has been surprising little research on the outcomes of transition in general (and ADHD in particular), but a recent scoping review identified six core components that could be used to evaluate interventions to support transition (Cleverley *et al*., 2018). These were transitional policy, tracking and monitoring, transition readiness, transition planning, transfer of care and completion of transfer. Similarly, a systematic review of transition for young people with various long term conditions in paediatric services suggested that preparation should commence in early adolescence but outcomes were better if transition was completed later, with 18 years recommended as the ideal age (Yassee *et al*., 2019).

There are some common barriers to the transition process in health care, some of which are relatively simple to address. A systematic review of transition from paediatric to adult care across varied conditions in the United States of America identified the following issues; *changing relationships, accessing adult practitioners, gaining funding, negative beliefs about adult care, lack of knowledge about the transition process* and *lack of self-management skills* (Gray *et al*., 2018). Similarly, a follow up study of young people in the UK with diabetes, cerebral palsy or autistic spectrum conditions found *appropriate* parent involvement, promotion of *health self-efficacy* and *meeting the adult team* before transfer were strongly associated with better outcomes (Colver *et al*., 2018). *Parental involvement* was also a predictor of successful transition from CAMHS to adult mental health services in the TRACK study (Singh *et al*., 2010). The children's services and adult services (CATCh-uS) study focused on transition in ADHD, and included semi-structured interviews with three groups of young people (before and after transition; plus those who dropped out of children's services and re-entered adult mental health services after a year or more); as well as parents and clinicians from CAMHS, paediatrics, adult mental health services and primary care (Janssens *et al*., in submission). Analysis indicated *the pivotal role of parents as advocates*, and a need to *balance the young person's participation in treatment decisions with the need to protect their interests according to their developmental capacity*. Developmental capacity may be particularly likely to be out of step with chronological age given the core impairments of ADHD (Coghill, 2017; Eke *et al*., 2019*a*). Echoing earlier work (Colver *et al*., 2018; Gray *et al*., 2018), participants in the CATCh-uS study emphasised two competing issues; *how prepared* the young person is for transition, and their *ability to manage their ADHD themselves*. Both are potential therapeutic targets, and the evidence-base would suggest that preparation should commence in early adolescence to provide time to develop self-management skills (Yassee *et al*., 2019).

Disengagement may occur before the upper age-boundary for the service if transition is not discussed, which contrasts with current common practice in many children's services, where the literature indicates little differentiation in the approach to young children or adolescents and poor awareness of adult provision (Buitelaar, 2017; Price *et al*., 2018). The CATCh-uS study revealed that many young people and parents lacked understanding that ADHD may persist into adulthood as well the common perception that medication was related to coping with school (Janssens *et al*., in submission). These were commonly cited reasons for dropping out of healthcare and suggest a need to support young people to *develop greater awareness of the impact of ADHD on their lives* and strategies to manage it (Buitelaar, 2017). Indeed, current guidelines would recommend *reassessment at the point of transition* as part of transition planning, which could prompt such discussions (Kooij *et al*., 2010; Young *et al*., 2016).

## ADHD and transition

There are also *condition specific barriers* to transition (Colver *et al*., 2018; Gray *et al*., 2018). For ADHD these are *lack of service provision, poor understanding or scepticism about ADHD* as a long term condition, and *insufficient knowledge* about the existence of adult ADHD services where these are available (Price *et al*., 2018; Janssens *et al*., in submission). In the CATCh-uS surveillance study, only 6% young adults with ADHD who needed and wanted to continue their ADHD medication experienced optimal transition at follow up; and only one fifth transferred successfully (Eke *et al*., 2019*a*). *Initial referral* (75% referred, 63% accepted) and *continuity of care after referral* (only 22% attended their first appointment at adult mental health services) were key weak points in the pathway, so should be targets for service improvement.

Lack of transitional service provision may lead to premature cessation of medication, inappropriate attendance by adults at children's services or discharge to primary care despite ongoing clinical need (Price *et al*., 2018; Tatlow-Golden *et al*., 2018; Janssens *et al*., in submission). Data from UK primary care suggest that even 5 years after national guidance recommended continued treatment for adults that require it, only 18% of young people prescribed medication for ADHD in their early teens continued to receive prescriptions beyond the age of 18 (NICE, 2008; Newlove-Delgado *et al*., 2019*a*). Of those who stopped their prescriptions, 7.6% had resumed them after the age of 20, and resumption was associated with referral to adult mental health services (Newlove-Delgado *et al*., 2019*b*). The process of re-accessing specialist mental health services was experienced as arduous, frustrating and lengthy (Price *et al*., 2018; Janssens *et al*., in submission).

We have very limited empirical evidence about how many young people require transition in relation to their ADHD, but that we have strongly suggests considerable under-provision. Prospective reports by consultant paediatricians and child psychiatrists across the United Kingdom and Southern Ireland suggest that between 270 and 599 per 100 000 17–19 year olds per year needed transition (Eke *et al*., 2019*a*). Given the increases in medication prescribing these figures should be expected to increase and will underestimate of the level of service provision required as inclusion in the study depended on needing and wanting continued medication. Many adults with ADHD want and could benefit from psychological support (Buitelaar, 2017; Coghill, 2017; NICE, 2018; Janssens *et al*., in submission) while triangulating these reports against a secondary data source suggested a high likelihood of incomplete case ascertainment (Eke *et al*., 2019*b*). These estimates should be taken as the lower limit of what is needed.

## What should adult ADHD services provide?

The focus on medication to the exclusion of other types of intervention is problematic (Janssens *et al*., in submission). Indeed, many of the factors highlighted that promote continuity of care involve education and the promotion of self-management. While many patients would welcome psychological support, practitioners report lacking the time or resources to deliver it (Janssens *et al*., in submission). There is little evidence currently that psychological therapy is effective for ADHD in childhood, but relatively few robust studies have been undertaken with adolescents and adults (Buitelaar, 2017). Motivational interviewing, cognitive behavioural approaches and mindfulness-based techniques might assist young people to improve their self-awareness as well as organisational, problem solving and decision-making skills, but need empirically testing (Buitelarr, 2017). As Professor Buitelaar asserts, we might be able to engage and support young people with ADHD using smartphone apps or games, provided evaluation demonstrated their effectiveness. Programmes that tailor support to the individual and include education, occupation and social issues may be more successful than medication provision alone (Embrett *et al*., 2016; Coghill, 2017). They could potentially be highly cost-effective, given the high rate of ADHD reported among prison populations (Young *et al*., 2018).

The difference in culture between child centred services, where parental involvement is assumed, to adult oriented services where parents were not necessarily included, causes problems for young people in transition and their carers (Singh *et al*., 2010; Price *et al*., 2018; Janssens *et al*., in submission). If parents are highly involved in supporting their child's access to health care, their exclusion from adult mental health services may lead to disengagement by default rather than intention (Colver *et al*., 2018; Janssens *et al*., in submission). There can, however, be tensions between the needs and wishes of the young adult and those of their carers, which all stakeholders need to negotiate carefully (Singh *et al*., 2010; Colver *et al*., 2018; Janssens *et al*., in submission). The balance can, and indeed should, change between parent-child dyads over time, and the management of this process should, if necessary, be a therapeutic target. In addition, there is an obvious issue for services to consider in terms of advocacy for young adults without parental support, such as those leaving the care system.

## Service organisation and transitional care models

Provision is strongly influenced by the historical development and funding processes (Crowley and Wolfe, 2013). Structural issues include the presence or absence of strong primary care, the availability of highly specialist centres of excellence, how specialist and primary care work together and whether primary and specialist care offer services for both adults and children, or physical and mental health. The extent to which health care is integrated with social and special educational services that many children with long term conditions need is also important (Crowley and Wolfe, 2013). The need for transition to adult services emerged with the shift from acute infections to chronic disease, while mental health services are particularly poorly resourced, and organised around episodes of care and severity (Crowley and Wolfe, 2013). There is huge variation in what is provided between and within countries, and whether the focus is restricted to ‘core’ mental health or broader needs (Certrano *et al*., 2020). Furthermore, paediatrics, CAMHS and adult mental health services are rarely all financed and administered within the same organisation, while training for professionals who work with children and adults often diverges at an early point. The resulting knowledge and cultural gaps combined with fragmentation of organisation, skills and knowledge-base as well as resources undermine collaborative working, which is essential to optimise transition (Coghill, 2017; Cortese and Barbui, 2017; Janssens *et al*., in submission). Stigma related to ADHD and adult mental health services may deter some young adults from transition (Young *et al*., 2016), which may be particularly salient for those initially treated within paediatrics as children. Indeed a lower proportion of young people transferred successfully from paediatric services than from CAMHS in the CATCh-uS surveillance study (Eke *et al*., 2019*a*). Moreover, social concerns about peers' evaluations may be particularly acute for adolescents (Buitelaar, 2017); the level of concern and considerations about stigma and disclosure should be a topic of discussion as part of transition preparation.

There have been a flurry of concerns about the provision of mental health care for young people, as well as for adolescents with long term conditions across high income countries (Crowley and Wolfe, 2013; McGorry *et al*., 2013). In Australia, ‘Headspace’ centres supplement traditional primary care for young people aged 12–25 years and offer easily accessible mental and physical health care, drug and alcohol services and access to vocational or educational advice as well as a public health remit that extends into schools and communities and includes on-line resources (McGorry *et al*., 2013). These centres have strong links to secondary mental health care centres that also focus on young people, particularly those with emerging severe mental illness and personality disorder. Similarly, the ‘Youthspace’ programme in the city of Birmingham is one of several UK-based examples of youth services that aim to provide easy access to specialist mental health care for young adults up to the age of 25; it includes a dedicated team for transition and specific consideration of ADHD (McGorry *et al*., 2013). While moving the upper age boundary to 25 avoids a break in provision at the maximal incidence for psychosis, eating disorders and personality disorder, it may merely postpone difficulties with transition for those with ADHD if the capacity of adult mental health services to work with this condition is not improved.

A systematic review identified three distinct but not mutually exclusive models of transitional care, all originating from the United States of America, as well as a lack of evidence to support their application (Nguyen *et al*., 2017). The framework for understanding mental health service utilisation classifies young people by their current needs and previous service use patterns to suggest a personalised approach to future care. The transition to independence model advocates for a transition worker to support individuals to plan their future care in relation to their needs. This is similar to the Transition Service Integration model, which incorporates the service context as well as individual needs to support future function. All models stress the need for broader services than mental health alone, and highlighted particular gaps in relation to sexuality, finance, environment and culture. We need evaluations of different models in a variety of locations to guide us as to which model is most effective in which context.

Despite the lack of evidence to support particular models of care in ADHD (Cortese and Barbui, 2017), there is much debate amongst stakeholders whether care for adults with ADHD can be adequately provided within *generic adult mental health services* or whether *dedicated specialist* services are preferable. There is also debate about *what constitutes a specialist service*? A Delphi study conducted about this issue in relation to eating disorders that concluded specialist services provide evidence-based interventions, must be multi-disciplinary, and staff working within the service must have a clear focus on, and expertise in, the focus condition (Petkova *et al*., in revision). The number of cases managed was also considered important but consensus was not achieved on how many were required to signify specialist expertise. An economic evaluation of specialist *v*. generic eating disorder services for young people suggested that specialist services did not produce better outcomes but as they worked with young people who had more severe difficulties, they were cost-effective depending on willingness to pay (Byford *et al*., 2019). It is intuitively plausible that a combination of regional highly specialist services to provide training, consultation and direct work with those with complex difficulties could compliment more widespread support in generic adult mental health teams, but proper evaluation is as desperately needed.

Adult ADHD services need to undergo similar development to that experienced by CAMHS and community paediatrics over the last three decades (Coghill, 2017). Clinical guidelines state that the following provision should be available for adults with ADHD: *transitional care, assessment and diagnostic services, drug titration, monitoring and review, and psychoeducation* (Nutt *et al*., 2007; Kooij *et al*., 2010; NICE, 2016, 2018; Young *et al*., 2016). Yet the research literature demonstrates that service provision remains highly variable between and within different countries, and that very few of those who need ongoing care for their ADHD make the transition to adult services, let alone experience anything that approaches optimal transitional care (Coghill, 2017; Eke *et al*., 2019*a*; Janssens *et al*., in submission).

We should be reassured that the epidemiological evidence points to continued under-recognition and under-treatment of children and young people in many countries (Ford *et al*., 2007; Coghill, 2017; Wang *et al*., 2017; Mandalia *et al*., 2018). But this also means that recent increases in the number of children prescribed ADHD related medication may continue and will logically be followed by an *increase* in the number of young adults who should transition. Service providers and commissioners should plan accordingly. As Professor Coghill argues, the training, experience and availability of professionals are more important than their qualifications or setting, but given how complex and variable the manifestations of ADHD can be, management by primary care without the support of specialist services may miss important comorbidities, even in ‘uncomplicated cases’ (Coghill, 2017). What is important for young adults and their carers is access to services that understand their condition and support to them to manage it, rather than where or by whom they are seen (Janssens *et al*., in submission). There is a desperate need for improved transitional and adult ADHD service provision, and now clear signals from research about what would improve continuity of care into adulthood; it is up to us all to implement and evaluate them.
